# Pleural Uptake Patterns in ^F18^Fluorodeoxyglucose-Positron Emission Tomography (FDG-PET) Scans Improve the Identification of Malignant Pleural Effusions

**DOI:** 10.3390/jcm12226977

**Published:** 2023-11-08

**Authors:** Samuel E. Cohen, Jaime Betancourt, Guy W. Soo Hoo

**Affiliations:** 1 Cedars-Sinai Medical Center, Los Angeles, CA 90048, USA; 2 West Los Angeles Veterans Affairs Healthcare Center, VA Greater Los Angeles Healthcare System, Los Angeles, CA 90073, USA; jaime.betancourt@va.gov (J.B.); guy.soohoo@va.gov (G.W.S.H.); 3 David Geffen School of Medicine at UCLA, Los Angeles, CA 90024, USA

**Keywords:** malignant pleural effusion, positron emission tomography, malignancy, FDG-PET, pleural disease

## Abstract

Background: The confirmation of malignant pleural effusions (MPE) requires an invasive procedure. Diagnosis can be difficult and may require repeated thoracentesis or biopsies. ^F18^Fluorodeoxyglucose-Positron Emission Tomography (FDG-PET) can characterize the extent of malignant involvement in areas of increased uptake. Patterns of uptake in the pleura may be sufficient to obviate the need for further invasive procedures. Methods: This is a retrospective review of patients with confirmed malignancy and suspected MPE. Patients who underwent diagnostic thoracentesis with cytology and contemporaneous FDG-PET were identified for analysis. Some underwent confirmatory pleural biopsy. The uptake pattern on FDG-PET underwent blinded review and was categorized based on the pattern of uptake. Results: One hundred consecutive patients with confirmed malignancy, suspected MPE and corresponding FDG-PET scans were reviewed. MPE was confirmed in 70 patients with positive pleural fluid cytology or tissue pathology. Of the remaining patients, 15 had negative cytopathology, 14 had atypical cells and 1 had reactive cells. Positive uptake on FDG-PET was noted in 76 patients. The concordance of malignant histology and positive FDG-PET occurred in 58 of 76 patients (76%). Combining histologically confirmed MPE with atypical cytology, positive pleural FDG-PET uptake had a positive predictive value of 91% for MPE. An encasement pattern had a 100% PPV for malignancy. Conclusion: Positive FDG-PET pleural uptake represents an excellent method to identify MPE, especially in patients with an encasement pattern. This may eliminate the need for additional invasive procedures in some patients, even when initial pleural cytology is negative.

## 1. Introduction

Pleural effusions are commonly encountered in patients with malignancy, occurring in an estimated 15% of patients, with a median survival of 3–12 months. This represents approximately 150,000 cases annually in the United States [1,2,3]. The presence of malignant cells in pleural fluid confirms metastatic disease [4,5]. For most types of cancer, evidence of metastasis to the pleural space limits available treatment options and indicates worse prognosis [4]. Traditionally, malignant pleural effusion (MPE) is diagnosed via the analysis of pleural fluid cytology obtained from thoracentesis or analysis of tissue from a pleural biopsy [4,6]. Thoracentesis is less invasive than pleural biopsy, but multiple thoracenteses may be required to improve diagnostic yield [4]. FDG-PET can identify areas of increased metabolic activity, which can be suggestive of malignancy—either primary or metastatic—but this may be confounded by localized inflammation, infection, or other non-specific causes of FDG uptake [7,8]. In the appropriate clinical context, FDG-PET can reveal sites of malignant involvement, especially in solid organs in the adult population. Additionally, FDG-PET can be used for identifying sites of hematologic malignancy such as lymphoma in both the adult and pediatric populations [9,10]. However, its role in those with pleural effusions has not been well defined.

There have been small retrospective studies suggesting that FDG-PET can be used for differentiating malignant from benign pleural effusions [11,12,13,14,15], and FDG-PET/CT could be even more accurate than FDG-PET or CT scans alone [16]. Another study found that the combination of cytology with FDG-PET/CT was better for identifying MPE in lung cancer than using any of these modalities individually [17]. Meta-analyses have concluded that FDG-PET scans could potentially be useful in identifying MPE, but the evidence across studies is not sufficient to endorse using FDG-PET for this purpose [18,19,20]. Another use for FDG-PET imaging, in the context of targeted genetic mutations, is related to the need for more tissue for molecular analysis after an initial cancer diagnosis [21,22]. FDG-PET can potentially be used to identify high yield locations for biopsy of the pleura.

Most reports incorporate a quantitative measure of uptake or the standardized uptake value (SUV), but our clinical experience suggests that qualitative analysis of FDG-PET images can identify characteristic patterns suggestive of malignant pleural involvement in patients with malignancy and pleural effusions. If validated, this finding could provide a non-invasive marker for pleural involvement in malignancy, which represents metastatic (M1a) disease in lung cancer staging. This has potential utility in the appropriate clinical circumstances, where this imaging may obviate the need for further investigation via thoracentesis, pleural biopsy or surgery. In the appropriate patient, abnormalities on FDG-PET scan may be sufficient to establish metastatic involvement of the pleura, thereby providing the confidence in clinical staging required to move onto treatment, expediting care that may have been delayed in time spent on confirmatory staging procedures. The following study represents our experience with FDG-PET scans in patients with cancer and suspected MPE.

## 2. Materials and Methods

This is a single-center study performed at the West Los Angeles Veterans Affairs Medical Center. We performed a retrospective analysis of adult patients with confirmed malignancy and suspected MPE who were evaluated in the hospital or Pulmonary Clinic from 2008 to 2021. All of the pleural effusions were deemed to be new in onset, with none of the patients having undergone any prior diagnostic procedure or pleurodesis. This is a consecutive case series of every patient in our hospital with suspected MPE who had a pleural study (thoracentesis and/or pleural biopsy), in addition to contemporaneous FDG-PET scans, who were included in the analysis. All but one patient underwent initial diagnostic testing with thoracentesis (one patient had a pleural biopsy as the initial test without thoracentesis). Further testing after thoracentesis was performed in some patients with either pleural biopsy or surgical biopsy, if determined to be clinically indicated. For patients with multiple thoracenteses, the sample used for analysis was the sample demonstrating malignancy or the one with the abnormal-appearing features. Pleural cytopathology results for each patient were then categorized, as reported by our Pathology Section, as follows: positive for malignancy, negative for malignancy, atypical cells present, or reactive cells present. The pleural fluid cell counts, including white blood cells (WBCs) with differential and red blood cells (RBCs) and pleural fluid lactate dehydrogenase (LDH), were also tabulated. 

**Figure 1:**
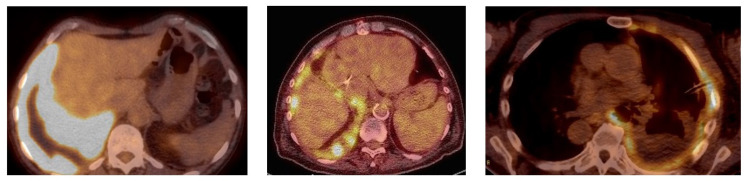
FDG-PET uptake patterns: (**left panel**) is encasement, (**middle panel**) is nodular and (**right panel**) is linear.

Two physicians blind to the results of the pleural cytopathology reviewed the uptake pattern of the FDG-PET studies. A third physician independently evaluated imaging and adjudicated the results in the event of discordance of interpretation. If FDG uptake was identified, it was characterized as having a linear, a nodular or an encasement pattern (Figure 1). The descriptions are self-explanatory, but there were cases where multiple patterns of uptake were present in the same patient. This was most commonly seen in patients with pleural nodules and the involvement of pleural lining, which is visually seen as the linear uptake. Each pattern of uptake was tabulated separately, even if present in the same patients. However, the abnormality was characterized as having an encasement pattern in patients for whom the FDG-PET uptake represented the involvement of the vast majority of the pleural surface, with the abnormal uptake appearing to wrap around the lung. This encasement pattern could have linear and nodular areas but was labeled as encasement given the overwhelming extent of involvement. While some patients had both patterns of nodular and linear uptake, encasement represented a more advanced degree of involvement and was mutually exclusive with nodular and linear uptake. The FDG-PET results were compared to the pleural cytopathology to assess the utility of this imaging modality for the confirmation of MPE.

### Data Analysis

Analysis of results was performed using MedCalc^®^, version 20.116 (2022 Brussels, Belgium). Basic descriptive statistics were derived from the above review and summarized in the table that accompanies this manuscript. Two by two tables were constructed, comparing pleural fluid results to FDG-PET scan results. These were dichotomous variables and allowed analysis using Fisher’s exact test and the calculation of sensitivity, specificity, positive and negative predictive values. The likelihood ratio and 95% confidence intervals were derived in a similar fashion. 

This study was reviewed and approved by our IRB, who waived the need for informed consent.

## 3. Results

A total of 100 consecutive adult patients were included in this cohort (94 male, 6 female), all of whom had confirmed malignancy (Table 1) and suspected MPE. All of the patients were veterans from the Greater Los Angeles VA Healthcare System. The average age was 67.0 ± 10.2 years. Out of the 100 patients identified for inclusion in this study, 99 initially underwent diagnostic thoracentesis; 1 patient had a pleural biopsy as the initial study rather than thoracentesis. Most patients only underwent one thoracentesis, but some had repeated procedures (2–3), and two patients had 5 and 6 thoracenteses, respectively. Additional pleural biopsies were performed in 14 patients, with a total of 15 pleural biopsies being performed. 

Values were tabulated from the specimen revealing malignancy or the specimen with the most abnormal reported cells. The pleural fluid was typically hemorrhagic in appearance (80,636 + 172,530 RBCs/μL; mean + SD), elevated WBC (2061 + 5099/μL), lymphocytic (51 + 29%) and exudative (serum LDH 590 + 986 U/L). The subgroup analysis of laboratory characteristics identified no significant differences between MPE and those with negative pleural fluid cytology. 

MPE was confirmed in 70 patients using either positive pleural fluid cytology or tissue pathology. Fifty-seven had malignancy identified via cytology. An additional thirteen patients were diagnosed via histology (one had pleural biopsy as the initial test, three had initial negative cytology, seven had initial atypical cytology and two had initial cytology with reactive cells). Of the 30 patients without outright positive cytopathology, 15 had negative cytopathology, 14 had atypical cells and 1 had reactive cells (Table 2).

**Figure 2:**
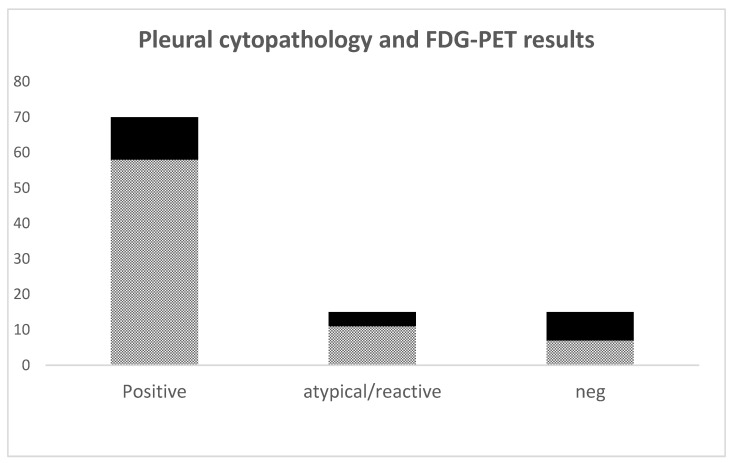
Results of pleural cytopathology and FDG-PET scans. Each bar represents results in each category, checkered boxes represent number of positive FDG-PET scans and black overlays represent negative FDG-PET scans.

Positive uptake on FDG-PET scans in the pleura was noted in 76 scans, with the uptake appearing nodular in 36, linear in 29 or encasement in 20, with some having more than one pattern (Table 2). No pleural uptake was seen in 24 scans. There was concordance of malignant cytology or history and positive FDG-PET in 58 of 76 (76%) patients (Figure 2). Eleven additional patients with positive FDG-PET scans had atypical cytologic results, representing a total of 69 patients with positive FDG-PET scans and malignant or abnormal cytology, representing 90.8% (69/76) of all positive FDG-PET scans. The remaining seven patients with positive FDG-PET scans had negative cytology.

Of the 24 patients with negative FDG-PET scans, 16 had atypical cytology and 8 had both negative cytology and negative FDG-PET scans. In cytologic and histologically confirmed MPE cases, the finding of pleural FDG-PET uptake represented a sensitivity of 82.9% and positive predictive value (PPV) of 89.2%, with specificity of 53.3% (Table 3). Including patients with cytology results of reactive or atypical cells, FDG-PET sensitivity remained about the same at 81.8%, with a PPV of 90.79% and specificity of 53.3% (Table 4).

It is notable that out of the 15 patients who underwent pleural biopsy, all were confirmed to have malignant pleural involvement. Their initial pleural fluid cytology was as follows: three negative for malignant cells, seven with atypical cells, two with reactive cells and two with positive for malignant cells. One patient did not undergo initial thoracentesis, and, therefore, no initial cytology was available for review. The abnormal cytologic findings coupled with abnormal FDG-PET imaging were often the impetus for pleural biopsy. Pleural biopsy resulted in an additional 12 patients being diagnosed with MPE who were not identified via cytology alone. One patient only had a pleural biopsy as the diagnostic study.

When analyzing FDG-PET results by the primary cancer, no significant differences in identifying MPE cases in primary lung (47/64 = 73%) or non-lung cancer primary (18/25 = 72%) were identified, but all patients with malignant mesothelioma had positive FDG-PET scans (11/11 = 100%). If mesothelioma is included as a primary lung malignancy, the FDG-PET yield is 77% (58/75).

The patterns of FDG-PET pleural uptake provided additional information on possible malignant pleural involvement. The encasement pattern was seen in 20 patients, and all 20 (100%) were diagnosed with malignant pleural involvement. In patients with a nodular pattern of FDG-PET uptake, 90% (36/40) had confirmed pleural malignancy. In patients with a linear pattern, 87.9% (29/33) were confirmed with pleural malignancy (Table 2). 

In those with encasement, eight patients were diagnosed with mesothelioma, and every one of the mesothelioma patients had FDG-PET uptake with the patterns of uptake listed in Table 2. Although suggestive of mesothelioma, other malignancies also present with encasement. Of twenty patients with encasement, eight (40%) were due to mesothelioma, with other malignancies comprising the other twelve (60%). Except for one patient with metastatic ovarian carcinoma, all of the other patients with the encasement pattern had primary lung cancer, i.e., either adenocarcinoma or poorly differentiated non-small cell carcinoma. The encasement pattern was strikingly predictive of malignancy, with a positive predictive value of 100% (Table 5). On the other hand, the absence of encasement did not preclude malignancy, but it seemed to exclude malignant mesothelioma as a diagnosis with a negative predictive value of 94.6% (Table 6).

## 4. Discussion

The diagnosis of MPE is one of the more challenging aspects in patients with malignancy. Confirmation establishes stage IV or metastatic disease (M1a) and eliminates curative surgical resection as a treatment option. The confirmation of malignancy may require several attempts, whether via thoracentesis or pleural biopsies [2]. It is well known that the identification of positive pleural fluid cytology may require repeated samples, and the acquisition of those samples may be tempered by the need for fluid to re-accumulate to permit sampling [1]. Other patient factors or preferences may also limit repeat sampling. Pleural biopsies are also limited by the sampling error or need for specialized resources, whether closed or thoracoscopically obtained. In addition, atypical cytology causes additional uncertainty and may further extend the time involved in management of these patients. Given the high index of suspicion for MPE, patients often undergo repeat sampling, and the confirmation of malignancy may require an extended time before confirmation, though confirmation may never occur given the advanced disease and/or co-morbidities. The duration involved with confirmatory studies is sure to contribute to uncertainty and delays in treatment, which, in turn, may adversely affect quality of life and patient outcomes.

It follows that the identification of MPE or a likely MPE using a reliable non-invasive marker such as FDG-PET scans could eliminate time delays encountered with confirmatory invasive sampling, which, in turn, would expedite cancer staging and treatment.

Our findings suggest that FDG-PET pleural uptake in patients with confirmed malignancy has a PPV just below 90% and can be a non-invasive tool for identifying MPE in the right clinical context (Table 3). The likelihood ratio is modest (1.78; 95% CI (1.02–3.08) but statistically significant. Including atypical cytology results, the PPV is just about 91%. This would suggest that in those patients with suspected malignant effusion, a FDG-PET scan with pleural involvement identifies about 90% of these patients with malignant effusions. A positive FDG-PET scan may be even more predictive of malignant involvement, as a diagnosis of a MPE often requires a second or third sample, which may not be possible. This is further borne out by our experience, with additional diagnoses of malignancy made by pleural biopsy, typically with the initial negative cytology. In other words, additional patients would have been identified with the pleural involvement on FDG-PET scans had they undergone additional pleural studies. 

Some cancers such as malignant mesothelioma are more likely to have pleural involvement, and FDG-PET seems especially useful in these cases [23,24]. Most of the cases of mesothelioma had an encasement pattern. However, it should be noted that of the 20 patients with an encasement pattern, the majority (60%) had a non-mesothelioma diagnosis. Except for one patient, primary lung cancer was the other malignancy most often associated with encasement on FDG-PET scans. This pattern of encasement and increased FDG-PET uptake was felt to be essentially pathognomonic for malignancy. While a common pattern in mesothelioma, other malignancies can present with a similar pattern of FDG-PET uptake. Encasement had a 100% positive predictive value for MPE. Conversely, encasement was the only FDG-PET pattern that was not seen in those patients with negative pleural fluid cytology. In those with a MPE, the absence of encasement virtually excluded malignant mesothelioma as the diagnosis with a NPV of 94.6%. The pattern of FDG-PET uptake may also be especially helpful in identifying MPE. Focusing on patients with pleural FDG-PET uptake, about 90% with either nodular or linear uptake were eventually identified as having MPE. The patterns of update generally involved an extensive portion of pleural surface, as depicted in the figure, and were typically adjacent to pleural effusions, as well as infrequently juxtaposed to areas of abnormal lung parenchyma. 

While pleural fluid cytology establishes malignant pleural involvement, there are many cases where the pleural fluid cells are atypical in appearance and, thus, a definitive diagnosis cannot be made. In these patients, confirming malignant involvement may require additional thoracenteses and cytologic exams or histologic confirmation via pleural biopsy, typically occurring several weeks or months after the initial sample is taken. This experience not only suggests that patients with pleural FDG-PET uptake and atypical cytology warrant continued efforts to confirm malignancy but also suggests that it may be a useful proxy for malignancy. Including these abnormal, but non-diagnostic, results in the analysis did not significantly change the sensitivity, specificity, PPV and NPV of a positive pleural FDG-PET scan. 

Our experience mirrors that of others evaluating FDG-PET scans for pleural involvement where there is often a high PPV [13] but modest NPV [25,26,27]. All of the patients in our series who had pleural biopsy were found to have malignant pleural involvement, despite 12 out of 15 initially having cytology without malignant cells, and only three were reported as negative. The others had cytologic atypia, but not cytologic malignancy, and, therefore, required additional biopsies. The yield on cytology from thoracentesis is modest and well described [4], and a confirmatory diagnosis often requires several repeated samples. These are often not obtained for a variety of reasons, so the presence of MPE is not able to be confirmed and likely underestimated in high-risk patients. In addition, many of these patients had clinical end-stage disease, and even if malignant pleural involvement was suspected, confirmatory studies were often deferred or never performed. 

The realities of the management of these patients provides an explanation of why investigations into the use of FDG-PET to identify MPE report relatively modest values for sensitivity and specificity. These values were calculated based on confirmed diagnoses of malignant pleural involvement, which may never occur due to poor patient status and clinically widespread disease. Patients with advanced clinical disease also emphasize the need for non-invasive markers of MPE, a role for which FDG-PET imaging seems to be best suited. In the past, confirming MPE was unlikely to have any impact on management in advanced cases of cancer, but with the advent of targeted chemotherapy and immunotherapy, more precise information on the extent of tumor involvement will help to monitor the response to therapy. It is in these situations that the findings of pleural involvement in FDG-PET may provide additional confidence in non-invasive staging and decisions on treatment. In these patients with advanced disease, less invasive interventions reduce the risk of complications associated with invasive investigations (thoracentesis or pleural biopsy). Conversely, patients with pleural effusions without pleural uptake on FDG-PET scans warrant further investigation, since paramalignant pleural effusion represents a lower stage of malignancy, in which case thoracentesis and pleural biopsies would be appropriate. 

Another possible limitation is the qualitative analysis of FDG-PET images. There is risk of variation in interpretation between physicians. A semi-quantitative analysis using SUVs could potentially reduce interpreter bias or differences related to the reading technique used, but there is no consensus threshold for a malignancy-defining SUV, and reported experience has also been variable. Touma and colleagues suggested that the semi-quantitative use of SUVs could be considered to identify MPE, but it only has modest accuracy [28]. Others suggest that either semi-quantitative use of SUVs [14,24,29,30] or a FDG-PET/CT scoring system [31] could be used to effectively differentiate malignant from benign pleural effusion. Duysinx and colleagues suggested that FDG-PET could be used to delineate intrathoracic vs. extra-thoracic malignancy, with intrathoracic malignancies having higher SUVs in comparison to extra-thoracic malignancies [24]. However, McAuley and colleagues found no statistical difference in SUVs when comparing benign and malignant effusions, though it was a relatively small study [32]. On the other hand, a meta-analysis by Porcel and colleagues suggested that the visual interpretation of FDG-PET may have better sensitivity than a semi-quantitative SUV approach [18]. While there seems to be merit to using objective cutoffs with SUV measurements, our experience using a qualitative approach and descriptions of patterns of uptake, in addition to clinical context, provided a high PPV in terms of identifying MPE via FDG-PET. The merits of this qualitative approach are best exemplified by an encasement pattern noted via FDG-PET imaging. Not only was encasement 100% predictive of malignancy, but it was also the only pattern for which no patient with the pattern had negative cytology.

There is concern that abnormal FDG-PET scan uptake is non-specific and may be confounded by associated inflammation. Much of this occurs in the context of infection or following pleurodesis [8]. None of the patients in this cohort had any other signs of infection or undergone pleurodesis. Pleural fluid characteristics manifested typical findings of MPE, specifically bloody, lymphocytic exudative effusions, which argue against an element of inflammation confounding these findings, providing additional support for the notion of malignancy causing pleural FDG-PET uptake.

## 5. Conclusions

FDG-PET is a valuable tool for identifying MPE in patients with malignancy. It is particularly effective in malignancies with frequent pleural involvement, such as malignant mesothelioma. In addition to uptake, patterns of encasement, nodularity or linear uptake were all associated with MPE. Overall, 100% of patients with an encasement pleural uptake pattern on FDG-PET scan were found to have MPE. With a positive predictive value of almost 90%, these findings of pleural uptake in an encasement, nodular or linear pattern upon performing a FDG-PET scan could negate the need for additional invasive diagnostic procedures in patients with a high clinical suspicion of MPE, even with negative pleural fluid cytology. If more tissue is needed for diagnosis or molecular analysis, regions of increased pleural uptake on FDG-PET can be targeted for biopsy. Additional prospective studies are needed to validate and standardize the interpretation of FDG-PET scans in suspected MPE cases.

## Figures and Tables

**Table 1:** Distribution of types of cancer in the cohort of suspected MPE.

Type of Malignancy	Number (Total = 100)
Lung	64
Mesothelioma	11
Aerodigestive tract	6
GI (not esophageal)	5
Breast	5
Lymphoma	4
Unknown primary	2
Ovarian	1
Prostate	1
Malignant melanoma	1

**Table 2:** Results of pleural fluid cytopathology, FDG-PET scan results and patterns of FDG-PET uptake.

Cytopathology Result	Total Cases	FDG-PET Pleural Uptake Present	Type of Uptake on FDG-PET Scan		
	*n* = 100	*n* = 76	Nodular	Linear	Encasement
Positive for malignant cells Cyto or histo	70	58	36	29	20
Cytology (+)	57	45	33	26	9
Pleural bx (+)	15	15	3	3	11
					
Cytology negative for malignant cells	15	7	4	4	0
Atypical or reactive cells present	15	11	4	3	4
					
Mesothelioma	11	11	2	2	8

**Table 3:** Comparison between pleural cytopathology results and FDG-PET scan results; atypical or reactive cytopathologies were not included.

	Pleural Cytopathology Positive	Pleural Cytopathology Negative (Excludes Atypical or Reactive Cytology)
FDG-PET Positive	58	7
FDG-PET Negative	12	8

FDG-PET uptake and positive cytopathology: sensitivity, 82.9%; specificity, 53.3%. Positive predictive Value: 89.2%; 95% CI: 82.7–93.5%. Negative predictive value: 40.00%; 95% CI: 24.8–57.3%. Positive likelihood ratio: 1.78; 1.02–3.08. Negative likelihood ratio: 0.32; 0.16–0.65. Fisher’s exact test: *p* = 0.0057.

**Table 4:** Comparison between abnormal pleural cytopathology (malignancy + atypical cells + reactive cells) and FDG-PET scan results.

	Pleural Cytopathology Positive + Atypical and Reactive Cells	Pleural Cytopathology Negative
FDG-PET Positive	69	7
FDG-PET Negative	16	8

FDG-PET uptake and positive/atypical cytopathology: sensitivity, 81.2%; specificity, 53.3%. Positive predictive value: 90.8%; 95% CI: 85.0–94.5%. Negative predictive value: 33.3%; 95% CI: 20.7–48.9%. Positive likelihood ratio: 1.74; 1.00–3.02. Negative likelihood ratio: 0.35; 0.18–0.67. Fisher’s exact test: *p* = 0.0076.

**Table 5:** Encasement pattern of FDG-PET scans and pleural malignancy.

	Malignancy	No Malignancy
FDG-PET scan encasement	20	0
FDG-PET scan negative, no encasement	16	8

Sensitivity, 55.5%; specificity, 100%. Positive predictive value: 100%. Negative predictive value: 33.3%; 95% CI: 25.76–41.78%. Negative likelihood ratio: 0.33; 95% CI: 0.13–0.88. Fisher’s exact test: *p* = 0.0049.

**Table 6:** Encasement of pleura pattern of FDG-PET scans and malignant mesothelioma.

	Mesothelioma	Malignancy, Not Mesothelioma
FDG-PET scan encasement	8	12
FDG-PET scan positive, no encasement	3	53

Sensitivity, 72.73%; specificity, 81.54%. Positive predictive value: 40%; 95% CI: 26.28–55.49%. Negative predictive value: 94.64%; 95% CI: 87.00–97.90%. Positive likelihood ratio: 3.94; 95% CI: 2.11–7.37. Negative likelihood ratio: 0.33; 95% CI: 0.13–0.88. Fisher’s exact test: *p* = 0.00066.

## Data Availability

Data are not publicly available but can shared upon request.
